# Genomic landscape and preclinical models of angiosarcoma

**DOI:** 10.1002/1878-0261.13744

**Published:** 2024-10-05

**Authors:** Annaleigh Benton, Bozhi Liu, Lauren E. Gartenhaus, Jason A. Hanna

**Affiliations:** ^1^ Department of Biological Sciences Purdue University West Lafayette IN USA; ^2^ Purdue University Institute for Cancer Research Purdue University West Lafayette IN USA

**Keywords:** angiosarcoma, genomics, rare cancer, sarcoma

## Abstract

Angiosarcoma is a cancer that develops in blood or lymphatic vessels that presents a significant clinical challenge due to its rarity and aggressive features. Clinical outcomes have not improved in decades, highlighting a need for innovative therapeutic strategies to treat the disease. Genetically, angiosarcomas exhibit high heterogeneity and complexity with many recurrent mutations. However, recent studies have identified some common features within anatomic and molecular subgroups. To identify potential therapeutic vulnerabilities, it is essential to understand and integrate the mutational landscape of angiosarcoma with the models that exist to study the disease. In this review, we will summarize the insights gained from reported genomic alterations in molecular and anatomic subtypes of angiosarcoma, discuss several potential actionable targets, and highlight the preclinical disease models available in the field.

## Graphical abstract

Angiosarcomas are a rare and aggressive cancer with a poor prognosis for patients. The genomic landscape of angiosarcoma can be highly complex and heterogenous; however, recent studies have identified some common features especially within anatomic and molecular subgroups. This review integrates these findings with available preclinical models and discusses actionable therapeutic targets for potential clinical advancements.

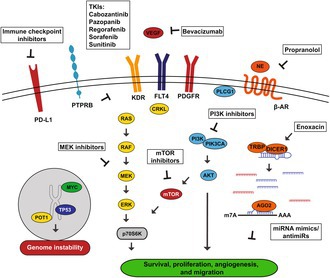

**Abbreviations**
AS: angiosarcoma
cKO: conditional knockout
HN‐AS: head and neck angiosarcoma
HSA: hemangiosarcoma
miRNA: microRNA
PDX: patient‐derived xenograft
TKI: tyrosine kinase inhibitor
TMB: tumor mutation burden
WES: whole exome sequencing
WGS: whole genome sequencing

## 1 Introduction

Angiosarcoma (AS) is an aggressive, highly metastatic endothelial cell cancer that develops in cells of the blood or lymphatic vasculature. The 5‐year survival rate of AS is only 30%, and patients with metastatic disease have a particularly poor prognosis, with a median survival time of only 12 months [1, 2]. AS can develop spontaneously or associated with prior radiation, chronic lymphedema, or exposure to toxic chemicals such as vinyl chloride [3]. Although a rare cancer, AS incidence rates are steadily increasing with over 1000 new cases per year now diagnosed in the US [4]. Tumors are generally treated with surgery, radiation, and chemotherapy. However, clinical outcomes have not improved in decades despite aggressive treatments [5].

Due to the rarity of AS, there are limited resources and research tools to understand the underlying biology. Thus, additional research is necessary to identify the drivers of the disease and possible actionable therapeutic targets. Several studies in relatively small independent cohorts have recently reported recurring mutations and genomic alterations in patient tumors. These studies have revealed several commonly altered genes, including *KDR*, *TP53*, *PTPRB*, and *PIK3CA* [3, 6, 7, 8, 9, 10, 11, 12, 13, 14]. Understanding these potential driver mutations in AS patients will enable the development of novel targeted therapeutics for personalized therapeutics. In this review, we will summarize reported genomic alterations in AS, dissect these genetic alterations based on anatomic localization subtype, review available models to study AS, and summarize current, emerging and prospective therapeutic strategies.

## 2 Common alterations in angiosarcoma

**Fig. 1:**
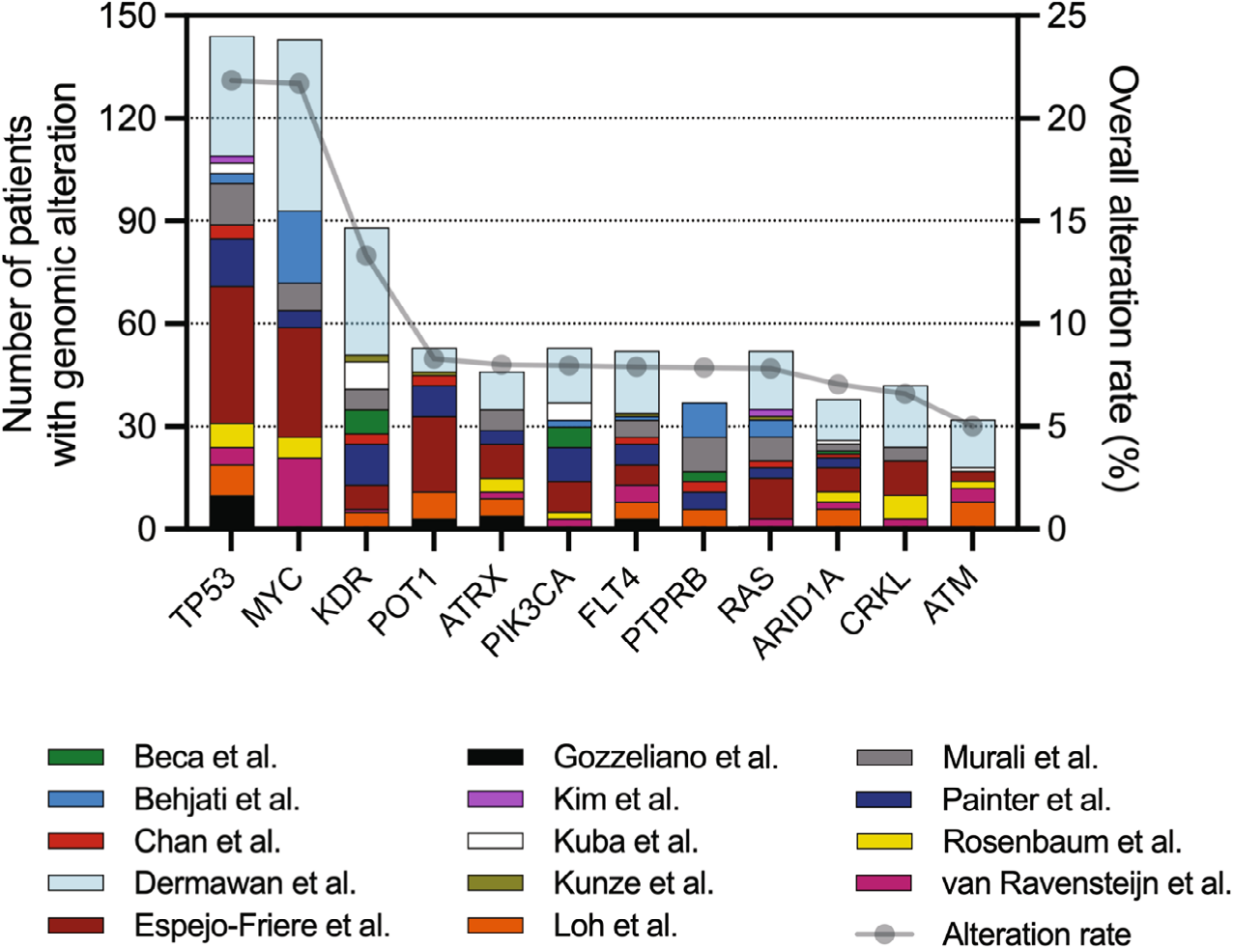
Genetic landscape of overall angiosarcoma patients. Stacked bar graph with the top 12 most frequent genomic alterations found in angiosarcoma. Genes are arranged according to overall alteration rate (right *y*‐axis, gray line) with the total number of alterations found for each gene (left *y*‐axis, stacked bar graph). The number of alterations and total number of samples sequenced for each gene are: *TP53* (144/659), *MYC* (143/659), *KDR* (88/661), *POT1* (53/638), *ATRX* (46/574), *PIK3CA* (53/665), *FLT4* (52/658), *PTPRB* (37/470), *RAS* (52/665 with *HRAS* (28/665), *NRAS* (16/665), and *KRAS* (8/665)), *ARID1A* (38/539), *CRKL* (42/637), and *ATM* (32/640).

Although studying genetic alterations in AS is informative, it is also important to note that other events, such as epigenetic and gene expression alterations, are also important drivers of oncogenic pathways. Nonetheless, several studies have recently performed whole genome, exome, or targeted sequencing to characterize common genomic alterations in AS (Table 1). Importantly these studies have identified recurrent alterations that are likely drivers of tumorigenesis. Though there are some similarities in the top mutated genes between studies (*TP53*, *MYC*, *KDR*, etc.), there is significant heterogeneity in the alteration frequencies, with some studies reporting highly mutated genes that do not appear in other cohorts. For example, high rates of *CSMD3* and *ERCC4* alterations have been reported in individual cohorts, but not found to be mutated in others [6, 7]. These variations may be due to the genetic heterogeneity of AS or differences in the patient populations. Additionally, due to the rarity of AS, cohorts are often relatively small and may not fully represent the overall population. To gain a more comprehensive understanding of the genetic landscape of AS it is beneficial to compare data from multiple independent studies. In compiling data from 14 studies, the most altered genes in AS are *TP53* and *MYC* followed by *KDR*, *POT1*, *ATRX*, *PIK3CA*, *FLT4*, *PTPRB*, *RAS*, *ARID1A*, *CRKL*, and *ATM* (Fig. 1).

**Table 1:** Studies that have reported genomic alterations in angiosarcoma patients. WES, whole exome sequencing; WGS, whole genome sequencing.

Study	Year	Number of patients	Platform
Beca et al. [10]	2020	10	Targeted, UCSF500 panel
Behjati et al. [12]	2014	3	WGS
Behjati et al. [12]	2014	8	WES
Behjati et al. [12]	2014	4	Targeted, 360 gene panel
Behjati et al. [12]	2014	24	Targeted, 28 gene panel
Chan et al. [9]	2020	18	WGS
Dermawan et al. [41]	2023	179	Targeted, MSK‐IMPACT 341 gene panel
Espejo‐Freire et al. [8]	2021	143	Targeted, 592 gene panel
Gozzellino et al. [13]	2023	24	RNA‐sequencing based variant analysis
Kim et al. [73]	2021	13	RNA‐sequencing based variant analysis
Kuba et al. [33]	2022	11	Targeted, MSK‐IMPACT 505 gene panel
Kunze et al. [49]	2014	6	Targeted 409 gene panel
Loh et al. [6]	2023	76	WGS
Murali et al. [11]	2015	34	Targeted, 341 gene panel
Painter et al. [26]	2020	36	WES
Rosenbaum et al. [3]	2022	26	Targeted, MSK‐IMPACT gene panel
van Ravensteijn et al. [7]	2022	50	Targeted, TSO500

High rates of *TP53* alterations are of no surprise as it is one of the most mutated genes in all cancers [15]. Indeed, TP53 functions as a tumor suppressor in endothelial cells as indicated in mouse and zebrafish models of AS [16, 17]. *MYC* amplification is commonly observed in secondary AS and alterations in angiogenic genes *KDR* and *PIK3CA* are more commonly observed in primary tumors. POT1 is also frequently altered and this protein is part of the shelterin complex, which plays a fundamental role in protecting chromosomal ends and telomere elongation [18]. POT1 alterations are causative for cardiac AS [18, 19, 20] and *POT1* mutant mice develop AS with 20% penetrance that is enhanced with additional loss of *TP53* [21].

## 3 Insights from angiosarcoma subgroups

Although there is clear heterogeneity in the genetic alterations in AS, classifying samples based on primary or secondary tumors (defined as AS associated with previous radiation therapy or chronic lymphedema) and anatomic location have provided evidence for specific alterations found in distinct AS groups. We will summarize these findings for these subgroups.

### 3.1 Head and neck angiosarcoma

Head and neck AS (HN‐AS) represents < 0.1% of all head and neck cancer, however, it has one of the highest lymph node metastasis rates of all soft tissue sarcomas [22]. HN‐AS has a low 5‐year survival rate of 26–51% [23]. In addition to low survival, it is often difficult to diagnose because it has a similar presentation to other conditions, such as hemangiomas, vascular malformations, and melanoma [24]. Recent studies have shown that UV‐associated HN‐AS tumors that are characterized by a high tumor mutational burden (TMB) are enriched for immune‐related signaling and display high tumor inflammation signatures [9]. This indicates that immunotherapies may be a viable therapeutic avenue, and indeed, HN‐AS patients have exceptional responses to immune checkpoint blockade therapies [25, 26]. Immunotherapies are often used in combination with other therapies to effectively combat tumors. By better understanding the common genetic alterations that drive HN‐AS tumors, these therapeutic targets may be identified and combined with immune checkpoint inhibitors.

**Fig. 2:**
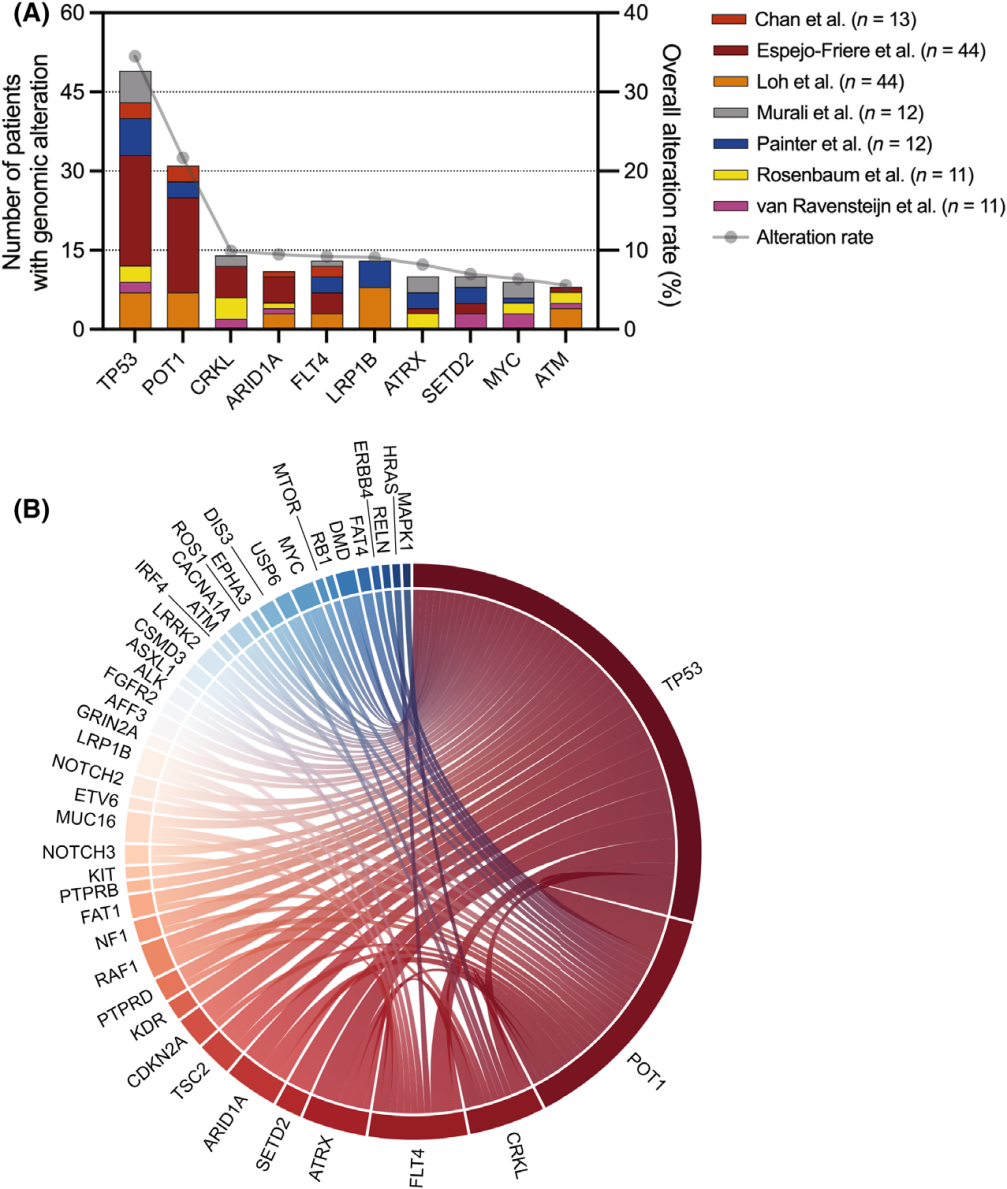
Genetic landscape of head and neck angiosarcoma. (A) Stacked bar graph with the top 10 head and neck angiosarcoma (HN‐AS) genomic mutations from seven independent studies. Genes are arranged by alteration rate (right axis, gray line) with total number of alterations found for each gene (left axis, stacked bar graph). The number of alterations and total number of samples sequenced for each gene are: *TP53* (49/142), *POT1* (31/143), *CRKL* (14/141), *ARID1A* (11/116), *FLT4* (13/141), *LRP1B* (13/143), *ATRX* (10/122), *MYC* (10/141), *SETD2* (10/143), and *ATM* (8/143). (B) Chord diagram generated by PlotAPI [27] of co‐occurring alterations involving *TP53*, *POT1*, *CRKL*, and *FLT4* observed in two or more HN‐AS patients.

We have compiled data from selected patients within the HN‐AS subtype and summarized the top 10 genetic alterations (Fig. 2A). *TP53*, *POT1*, and *CRKL* were the most prevalent genomic alterations, followed by *ARID1A*, *FLT4*, *LRP1B*, *ATRX*, *SETD2*, *MYC*, and *ATM*. *TP53* remains the top mutated gene in HN‐AS as it is in overall AS. However, the rate in HN‐AS is even higher (35% vs. 22%) compared to the frequency in AS overall. *TP53* also highly co‐occurs with other mutations in HN‐AS and is the dominant co‐occurring mutation [27] (Fig. 2B). The incidence of *POT1* alterations is also nearly tripled in HN‐AS compared to overall AS whereas *MYC* amplification is less frequent. *FLT4* is the sole angiogenesis‐related gene in the top 10 which further exemplifies unique features of HN‐AS compared to other subgroups. Thus, therapeutic inhibition of these pathways altered in HN‐AS combined with immunotherapies could be an effective strategy for these patients.

### 3.2 Primary breast angiosarcoma

Breast AS represents < 1% of all breast cancers and < 5% of all sarcomas [28]. There are two subtypes of breast AS, primary and secondary breast AS. Primary breast AS develops spontaneously, whereas secondary breast AS is associated with prior radiation therapy [29, 30, 31]. Primary breast AS typically occurs within the breast parenchyma with both cutaneous and subcutaneous involvement [29]. The median age of primary breast AS patients is around 40 years of age [29, 31]. According to a large cohort study, the 5‐year and 10‐year survival rates of primary breast AS patients are 49% and 38%, respectively [32]. Currently, the risk factors of primary breast AS are unknown, and its genetic features have not been well characterized.

**Fig. 3:**
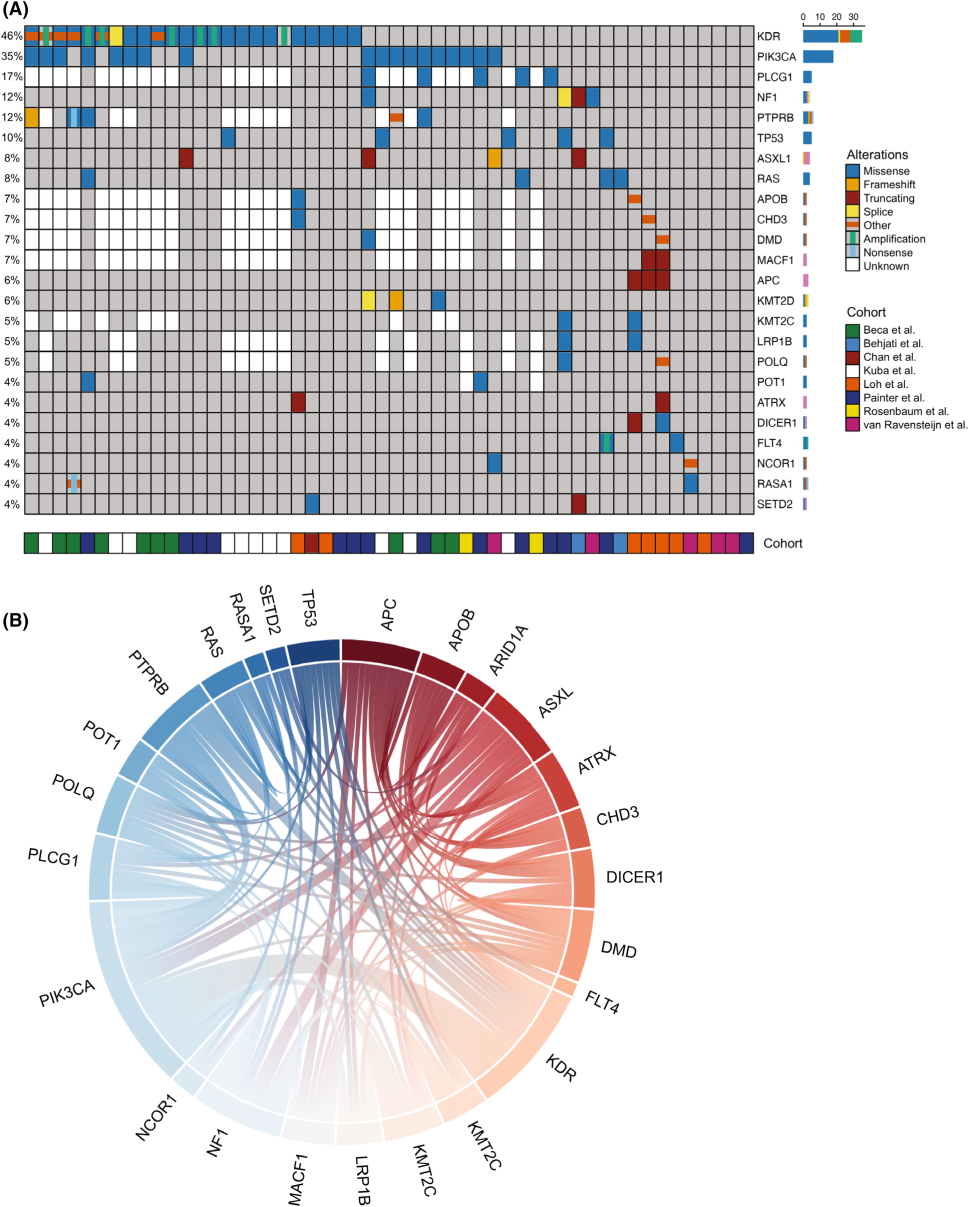
Genetic landscape of primary breast angiosarcoma. (A). Oncoprint with genomic variants and copy number changes in frequently altered genes in primary breast angiosarcoma (AS). ‘Other’ includes non‐frameshift indel, in‐frame deletion, multi‐hit mutations, and biallelic inactivation of tumor suppressor genes. (B) Chord diagram generated by PlotAPI [27] of frequent co‐occurring alterations found in two or more primary breast AS patients.

However, eight studies [3, 6, 7, 9, 10, 12, 26, 33] and a total of 52 samples with clear identification of primary breast AS and genomic sequencing data are available. Of note, all patients are female. The top five altered genes are *KDR* (46%), *PIK3CA* (35%), *PLCG1* (17%), *NF1* (12%), and *PTPRB* (12%). Interestingly, alterations in *MYC*, *TP53*, and *FLT4*, which are the top altered genes in other subgroups of AS, are less frequent in primary breast AS (Fig. 3A). Instead, frequent alterations in angiogenesis‐related genes and PI3K/AKT pathways are observed. The impact of *PIK3CA* mutations was recently noted in a small cohort analysis indicating patients with *PIK3CA* mutations correlate with worse prognosis [33].

Altered genes that are reported in more than one tumor sample were examined by a chord plot (Fig. 3B). *PIK3CA* mutations often co‐occur with alterations in *KDR* or *PTPRB*. Additionally, *KDR‐PTPRB*, *PIK3CA‐ASXL1*, *NF1‐ASXL1*, *KMT2D‐PIK3CA*, and *PLCG1‐PIK3CA* co‐occurring alterations are also frequently observed. *PLCG1* and *KDR* are mutually exclusive in primary breast AS and this phenomenon was also observed in general AS patient [34, 35]. Of these interactions, *KDR* and *PIK3CA* co‐occurrent alterations correlate with poor prognosis indicating their importance in primary breast AS [33]. This finding should be further evaluated in large patient cohorts. Nonetheless, this highlights the potential in genomic testing and utilizing molecular features in stratifying patients and potentially directing targeted therapies to specific pathways and patient subgroups.

### 3.3 Secondary angiosarcoma

Secondary AS arises in association with prior radiation treatment or chronic lymphedema, known as radiation‐associated AS or Stewart‐Treves syndrome respectively [36]. Additionally, certain chemicals, such as thorium dioxide and vinyl chloride, are risk factors of AS [37]. Breast cancer patients treated with radiation therapy are at risk of developing secondary breast AS, which has distinct clinical features compared to primary breast AS. It typically occurs in the cutaneous and/or subcutaneous layer of the skin within the irradiated area and is often associated with purple‐blue skin discoloration [31]. Patients tend to develop secondary breast AS at an older age [38], with a median latency period of over 10 years between the initial treatment and the development of secondary AS [39]. The 5‐year survival rate of secondary breast AS is 42% [32, 40]. van Ravensteijn et al. [7] reported *MYC* amplification in 100% of Stewart‐Treves AS and 92% of radiation‐induced AS. Additionally, they found *FLT4* amplification in 31% of radiation‐induced AS cases, which always co‐occurred with amplified *MYC*. Furthermore, mutations in the DNA damage response pathway were found in more than half of the secondary AS cases. Lesluyes et al. [14] noted frequent loss of *CDKN2A* and *CDKN2B* and recurrent *MYC* amplification in radiation‐induced sarcomas compared to sporadic sarcomas. Frequent *MYC* and *FLT4* alterations in radiation‐induced AS was also reported in Chang et al. and Dermawan et al. [40, 41]. This demonstrates that some genetic alterations are commonly associated with radiation‐associated AS. Radiation‐associated AS occurrence is steadily increasing as radiotherapy for breast carcinomas has increased [4]. Although the risk of AS development is relatively low, alternative breast conservation therapies or optimized radiotherapy techniques should be investigated to minimize this risk further [4].

### 3.4 Cardiac angiosarcoma

Cardiac tumors are extremely rare with an autopsy incidence of 0.056–1.23% [42]. Cardiac AS specifically accounts for approximately 7.3–8.5% of all cardiac tumors [43, 44]. In one cohort of AS patients, 5% of primary AS tumors were derived from the heart [26]. Although the prognosis of AS patients is generally poor, the prognosis for cardiac patients is particularly dire. Without surgical resection, the average survival of patients with cardiac AS is just 3 months [45]. Additionally, given the low incidence of cardiac AS, the causes are not well understood. However, some studies have indicated certain patient characteristics that seem to be correlated with higher rates of disease. One study exploring primary malignant cardiac tumors found that black patients more commonly present with cardiac AS [46] and it is also more common in males than females with a ratio of 2–3 : 1 [47].

In five studies that identified gene mutations in patients with cardiac AS, the commonly mutated genes are *KDR*, *POT1*, *PLCG1*, and *LRP1B. KDR* mutations were found in 6 out of the 18 cardiac AS patients [6, 7, 26, 48, 49]. *KDR* has been noted previously with activating mutations in 10% of AS cases [50]. *PLCG1* mutations were the next most frequent with one study identifying 3 of 10 patients harboring the *R707Q* mutation in the autoinhibitory SH2 domain [49]. Additional studies have found *POT1* mutations associated with familial cardiac AS in Li‐Fraumeni‐like syndrome families [19, 20]. Li‐Fraumeni syndrome is an autosomal dominant disorder that increases the risk of developing multiple cancers including sarcomas [19, 51]. POT1 is a subunit of the shelterin complex, that protects chromosomal ends and regulates telomere length [18]. The p.R117C variant of *POT1* is associated with abnormally long telomeres and promotes cardiac AS [19, 20]. Furthermore, mice harboring this allele also develop cardiac AS [21]. Given their aggressive nature and the rarity of cardiac AS, there is not a defined treatment approach. Following surgery, radiation and an anthracycline‐based therapy is recommended [52]. In general, surgical resection provides patients with cardiac sarcomas the best outcomes, but this is not an option for all patients due to the delicate surrounding tissue and organs [53], emphasizing the need for better therapeutic strategies.

## 4 Preclinical models of angiosarcoma

Given the poor outcomes, unclear optimized treatment strategies, and lack of targeted therapies in AS, it is essential to model the disease and advance our understanding of the drivers of tumorigenesis. Due to the rarity of AS, models and research tools are lacking compared to other cancers. However, through the efforts of many groups, these resources are becoming more available and will be summarized below.

Angiosarcoma like other tumors constitute a complex extracellular environment (ECM) that is difficult to recapitulate *in vitro*. These ECM interactions seem particularly important for AS since many labs have faced challenges generating AS cell lines and patient‐derived xenograft (PDX) models [54, 55]. Furthermore, recent studies indicate endothelial cells are considerably sensitive to contextual transcriptional changes especially in cell culture conditions [56]. Nonetheless, there are some human cell lines and reports of short‐term culturing AS cells, providing research tools for practical hypothesis testing (Table 2). Interestingly, the majority of the reported human cell lines have been generated from scalp AS tumors and likely harbor UV‐damage signatures. Indeed, Loh et al. [6] recently reported high mutation burdens of > 7 mutations per coding megabase in the ASM, ISO‐HAS, and MOLAS cell lines. Of note, the ASM cells are characterized by mutations in *TP53*, *ARID1A*, and *FLT4*; the ISO‐HAS cells with a *TP53 mutation*; and the MOLAS cells with a *POT1* mutation. Thus, these lines are great models of scalp AS. Future work should focus on generating cell lines from other anatomic and molecular subtypes. Additionally, increased “omics” characterizations of the other cell lines would be beneficial for the field, as there is limited information on the genomic alterations and few gene expression studies in the cell lines to date [6, 57].

**Table 2:** Angiosarcoma cell lines and PDX models. AS, angiosarcoma; HSA, hemangiosarcoma; PDX, patient‐derived xenograft.

Cell line/PDX	Species	Origin	Xenograft	Reference
AS‐02	Human	Primary culture from a 71‐year‐old Caucasian female with secondary breast AS (radiation‐induced AS)	–	Azzariti et al. [140] and Porcelli et al. [141]
AS‐5	Human	Cell line derived from a primary AS of the thigh from 82‐year‐old male patient	–	Italiano et al. [142]
AS‐M	Human	Cell line derived from a cutaneous AS of the scalp from 80‐year‐old patient	No	Krump‐Konvalinkova et al. [143]
HAMON	Human	Cell line from an 81‐year‐old recurrent Scalp AS, tumor implanted in nod/scid and cell line generated from xenograft	No	Hoshina et al. [144]
ISO‐HAS	Human	Cell line derived from a metastatic anterial‐auricular lesion from a primary parietal‐scalp 84‐year‐old male	Yes	Masuzawa et al. [145]
KU‐CAS3	Human	Cell line from regional metastatic scalp AS sample from 73‐year‐old male	Yes	You et al. [58]
KU‐CAS5	Human	Cell line from scalp AS from 78‐year‐old female patient	Yes	You et al. [58]
MO‐LAS	Human	Cell line derived from 77‐year‐old male with metastatic lymphangiosarcoma of the scalp, cell line established from pleural fluid cells	–	Masuzawa et al. [146]
PCB‐011	Human	Primary cell culture from 64‐year‐old female AS patient	–	Dr Charles Keller and Brashears et al. [147]
RT‐AS5	Human	Patient‐derived xenograft model from radiation‐induced breast AS patient	Yes	Versleijen‐Jonkers et al. [59]
ADC106	Mouse	Cell line generated from an *aP2‐Cre; Cdkn2a* ^*cKO*^ *; Dicer1* ^*cKO*^ AS tumor	Yes	Hanna et al. [66]
*Cdh5‐CreER* ^*T2*^ *; Trp53* ^*cKO*^	Mouse	Cell line from *Cdh5‐CreER* ^*T2*^ *; Trp53* ^*cKO*^ tumors	No	Salter et al. [148]
Hepatic AS	Mouse	Cell line from *Notch1* knockout hepatic AS tumor	Yes	Rothweiler et al. [149]
ISOS‐1	Mouse	Cell line from 84‐year‐old male scalp tumor, tumor tissue implanted in SCID mouse, then mouse cell line generated from xenograft	Yes	Masuzawa et al. [150]
*Pdgfrb‐Cre; Trp53* ^*R172H/R172H*^	Mouse	Cell lines from *Pdgfrb‐Cre; Trp53* ^*R172H/R172H*^ *tumors* (noted loss of endothelial characteristics)	Yes	Salter et al. [148]
SVR	Mouse	Subclone of the MS1 immortalized pancreatic islet endothelial cell line transformed by oncogenic Hras	Yes	Arbiser et al. [151]
Tsc1^iΔEC^	Mouse	Primary cell cultures from Tsc1^iΔEC^ lymphangiosarcomas	Yes	Sun et al. [67]
Cindy	Canine	HSA cell line from surgical sample	–	Urbasic et al. [85]
COSB	Canine	Derivative of the SB‐HSA cell line	Yes	Kim et al. [81]
Dal‐4, DD‐1, Chad‐G4, Chad‐G6, Chad‐B7, Chad‐G8, Chad‐P9, Emma	Canine	HSA cell lines generated in culture from surgical samples	–	Fosmire et al. [78]
DEN‐HSA	Canine	Renal HSA cell line	–	Thamm et al. [76]
DHSA‐1426	Canine	Splenic HSA cell line that consistently grows as xenograft	Yes	Kim et al. [86]
JHE, JLI, JLU, JSP	Canine	Cell lines established from HSA tumors in heart, lung, liver, spleen from same animal	–	Kim et al. [81]
JOEY‐HSA, JOURNEY‐HSA, TUCKER‐HSA	Canine	HSA cell lines generated in culture from surgical samples	–	Tamburini et al. [82]
JuA1, JuB2, JuB4, Re12, Re21, Ud2, Ud6	Canine	Cell lines generated from 3 xenograft HSA tumors from spontaneous HSA of liver, heart, or spleen	Yes	Murai et al. [87]
MOCHA‐HSA, FROG‐HSA	Canine	HSA cell lines generated in culture from surgical samples	–	Lamerato‐Kozicki et al. [84]
SB‐HSA	Canine	Cutaneous HSA cell line established from xenograft	Yes	Akhtar et al. [80]

In addition to cell line development, significant efforts have been made to generate *in vivo* AS models. However, similar challenges have been faced with these approaches. Of the human cell lines reported only a few of them are able to grow as xenografts in immunodeficient mice [58]. Additionally, tumors directly implanted into mice for PDXs have had limited success. One study reported 0 of 5 implanted AS tumors established a PDX [54]. A recent study was able to generate a single PDX out of four radiation‐associated secondary breast AS tumors [59]. Genomic analysis of this PDX revealed *MYC* and *FLT4* amplification as well as a likely pathogenic *FANC1* deletion. *MYC* and *FLT4* are co‐amplified in 25% of secondary breast AS, thus this provides a fantastic model for secondary breast AS tumors. This is of particular interest as this subtype is increasing in incidence due to the rise in breast conservation radiotherapy among breast cancer patients [4].

Genetically engineered mouse models (GEMMs) have been valuable and provided insights into our understanding of tumor cell origins as well as the necessary, sufficient, and cooperative contributions of individual genes in cancer biology. Indeed, several GEMMs spontaneously develop AS (Table 3). Some of these models are quite penetrant and specific with only AS development. Unsurprisingly, *Trp53* alterations in endothelial cells is a common driver of AS either alone or in combination with the loss of additional tumor suppressors such as *Pten* and *Ptpn12* [60]. Additionally, in zebrafish, Tr*p53* deletion results in AS, malignant peripheral nerve‐sheath tumors, germ cell tumors, and leukemia development [16, 17]. In humans, a *POT1* Li‐Fraumeni like mutation is responsible for a familial predisposition to a spectrum of tumors including sarcomas and particularly cardiac AS. Studies of this mutation in mice indicate that germline (*Pot1a* ^*R117C*^) mice develop AS including cardiac AS and rarely other tumors. This phenotype can be amplified with combined *Trp53* loss [21].

**Table 3:** Preclinical genetically engineered mouse models of angiosarcoma. AS, angiosarcoma; LAS, lymphangiosarcoma.

Model	Phenotype	Reference
*aP2‐Cre;Cdkn2a* ^*cKO*^ *;Dicer1* ^*cKO*^ or *aP2‐Cre;Tsc1* ^*cKO*^ *;Dicer1* ^*cKO*^	Broad anatomic distribution AS in 100% by 28 weeks	Hanna et al. [66]
*aP2‐Cre;Cdkn2a* ^*cKO*^ *;LSL‐Kras* ^*G12D*^	100% AS and other tumors rarely by 7 weeks	Drummond et al. [68]
*aP2‐Cre;Dicer1* ^*cKO*^	Broad anatomic distribution AS in 100% by 58 weeks	Hanna et al. [126]
*Cdh5‐CreER* ^*T2*^ *;p53* ^*cKO*^	Broad anatomic AS in 100% by 57 weeks	Salter et al. [148]
*Cdk4* ^*R24C/R24C*^ or *Cdk6* ^*R31C/R31C*^	AS (58–38% respectively) and several other tumors	Rodríguez‐Díez et al. [70]
*Darpp32‐Cre;Tsc1* ^*cKO*^	Paw AS in 100% by 6 weeks and renal cystadenomas	Leech et al. [152]
*End‐Scl‐Cre‐ER;Tsc1* ^*cKO*^	Cutaneous tail/paw LAS in nearly 100% by 40 weeks, other vascular tumors	Sun et al. [67]
*GFAP‐CreER;p53* ^*cKO*^ *;Pten* ^*cKO*^ *;Ptpn12* ^*cKO*^	AS Tumors nearly 100% by 28 weeks	Chadwick et al. [60]
*Ink4a/Arf* ^*−/−*^	30% AS by 15 weeks depending on strain	Yang et al. [153]
*Ink4c* ^*−/−*^ *;Ink4d* ^*−/−*^ *;p53* ^*−/−*^	AS (63%) and other tumors by 30 weeks	Zindy et al. [55]
MuLE lentiviral *Hras* ^*G12V*^ with *Cdkn2a* or Tr*p53* shRNA	88% AS in CB17 mice by 4 weeks	Brandt et al. [154]
*Mx‐Cre;Notch1* ^*cKO*^	Hepatic AS in 86% by 50 weeks	Dill et al. [69]
*Pdgfrb‐Cre;TP53* ^*R172H/R172H*^	Several tumor types with AS in 75% by 26 weeks	Salter et al. [148]
*Pot1a* ^*R117C/+*^	14% AS in thorax cavity and heart, rare other tumors by 150 weeks	Martínez et al. [21]
*Prrx1‐Cre;Tsc2* ^*cKO*^	Paw AS/LAS in nearly 100% by 40 weeks, other vascular tumors and renal cystadenomas	Klover et al. [155]
*Tcl1b* ^*Tg*^	AS of the intestinal tract in 2/2 mice within 1 year of age	Hashimoto et al. [156]
*Tie2‐CreER;p53* ^*cKO*^ *;Pten* ^*cKO*^ *;Ptpn12* ^*cKO*^	Broad anatomic distribution of AS	Chadwick et al. [60]
*Tie2‐Cre;Trp53* ^*cKO*^	62% AS and other tumors within 50 weeks	Ghahremani et al. [157]
*Trp53* ^*R172H/−*^	Many tumors and AS in 62% of mice by 32 weeks	Olive et al. [158]
*Trp53* ^*−/−*^ *;p16Ink4a* ^*−/−*^	56% AS and other tumors	Sharpless et al. [159]
*Tsc1* ^*+/−*^	Paw AS (~10%), renal cystadenomas, lung adenomas, liver hemangioma by 60 weeks	Kwiatkowski et al. [160] and Kobayashi et al. [161]
*Tsc2* ^*+/−*^	Rare paw AS, renal cystadenomas, lung adenomas, liver hemangiomas by 60 weeks	Onda et al. [162] and Kobayashi et al. [163]

Mouse models have also indicated the importance of tumor suppressing microRNAs (miRNAs) in AS. Our lab has found that conditional deletion of *Dicer1* with *aP2‐Cre* leads to AS development with 100% penetrance. Recent work by us and others have provided evidence for some key miRNAs lost in AS [61, 62, 63, 64, 65]. We have also found that *Cdkn2a* or *Tsc1* loss cooperates with *Dicer1* deletion to accelerate tumor development [66]. In agreement, several groups have demonstrated the importance of the mTOR pathway in AS [60, 67]. Additionally, oncogenic *RAS* while mutated in a smaller subset of AS ranging from 7% to 25%, also drives AS in mice [68]. Finally, studies of *Notch1* and mutant *Cdk4/6* are also implicated in GEMMs [69, 70].

In addition to mouse and zebrafish preclinical models, AS otherwise known as hemangiosarcoma (HSA) occurs with a much higher incidence in dogs than in humans [71]. This provides a unique opportunity to take advantage of comparative oncology and understand the common and unique features of AS and HSA. Indeed, some of the same genetic alterations are found in canine HSA such as *TP53*, *PIK3CA*, *RAS*, *CDKN2A*, and *KDR* [72, 73, 74]. One notable exception is a lack of high tumor mutation burden (TMB) in HSA, suggesting canine tumors may not respond to immunotherapies like human AS tumors with high TMB [73, 74, 75]. Recently, novel chromosomal translocations and gene fusions were reported to be associated with TP53 mutant HSA and AS [73]. Additionally, an extensive collection of canine HSA cell lines and PDXs have been established by several groups [76, 77, 78, 79, 80, 81, 82, 83, 84, 85]. Some of the cell lines also grow well as xenografts in mice providing valuable *in vivo* resources [80, 81, 86, 87]. Recently, studies on the generation of canine HSA cell lines with CRISPR engineered *PIK3CA* activating mutations indicate altered oncogenic RAS, MAPK, and PI3K pathways as well as altered cytokine signaling [88]. In addition, these mutant HSA cells serve as a model to study therapeutic resistance to PI3K inhibitors and provide a valuable platform for testing therapeutic combinations for *PIK3CA* mutant AS in future studies [89].

The therapeutic use of the non‐specific beta‐adrenergic receptor antagonist, propranolol, in AS is another example of the feedforward knowledge sharing possible with comparative oncology [90]. Propranolol is used clinically in benign pediatric hemangiomas and recent clinical case reports indicate some AS tumors are also responsive to propranolol [91]. Further work in canine HSA cell lines demonstrates effective growth suppression with propranolol [91, 92]. Thus, a phase 1 clinical trial in canine splenic HSA has been conducted and found potential enhancement of long‐term survival in some canine patients [93]. Additionally, a recent human clinical trial testing propranolol monotherapy found modest clinical benefit in two out of 14 patients suggesting propranolol in combination with other therapies may be more efficacious [94].

Future studies with other novel therapies should similarly take advantage of these comparative oncology approaches. With the current available models, some of the top mutated genes in AS are represented. However, models with amplification of *CRKL*, which is one of the top 3 mutated gene in HN‐AS, are still lacking. Additionally, models representing primary breast AS with altered *KDR*, *PIK3CA*, and *PLCG1*, have also not been evaluated. In the future, these additional models may lead to a better understanding the underlying biology of different subtypes of AS.

## 5 Therapeutic implications of genetic alterations

**Fig. 4:**
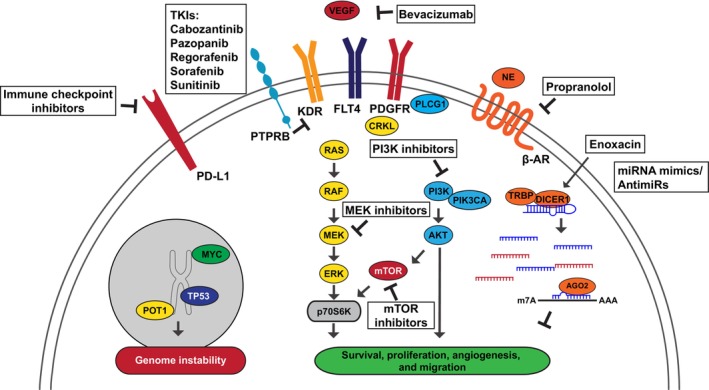
Potential targeted therapies for angiosarcoma. Altered pathways and potential therapeutic opportunities in angiosarcoma.

For advanced AS, systemic therapy is required. However, there is currently no standard first‐line therapy, and treatment options are limited and offer only modest benefits [37, 95]. Due to the rarity of AS, it can be difficult to enroll an adequate number of patients into clinical trials to achieve the necessary statistical power. Furthermore, the heterogeneity in presentation, histopathology, and molecular characteristics of AS poses difficulties in standardizing treatment approaches and patient selection criteria across clinical trials. Despite these obstacles, it is of critical importance to strive for novel therapeutic approaches to improve patient survival and clinical trial accrual is encouraging despite the rarity of AS [96]. The commonly altered genes in AS and its subtypes highlight many therapeutic targets already being pursued, and other potential candidates that may be considered in specific subtypes of AS (Fig. 4).

Vascular endothelial growth factor receptors (VEGFRs) have been among the most studied as potential therapeutics for AS. KDR and FLT4 (VEGFR‐2 and VEGFR‐3, respectively) are tyrosine kinases in endothelial cells, and are among the top altered genes in AS. VEGFR‐related signaling pathways are involved in the survival, proliferation, migration of endothelial cells and the development of new blood vessels, which can be mediated through PI3K/Akt pathway activation [97, 98, 99, 100, 101]. Despite the high activity of VEGF‐VEGFR pathway in AS, clinical trials testing therapeutics designed to target this pathway have had disappointing results [37]. Because *KDR* (VEGFR2) alterations lead to VEGF independent constitutive activation of VEGFR2 in many AS patients, VEGF ligand targeting molecules such as bevacizumab may not be ideal in these patients [50]. Indeed, bevacizumab trials have not indicated broad responses [102, 103]. Instead, small molecule multi‐tyrosine kinase inhibitors (TKIs) may be better suited for patients with *KDR* alterations. Pazopanib, sorafenib, sunitinib, regorafenib, and cabozantinib have been tested in several AS patient cohorts. The benefits are broadly modest but have shown some promise [37, 104, 105]. Regorafenib has shown some benefit in metastatic or unresectable AS [102]. Excitedly cabozantinib combined with the immune checkpoint inhibitor, nivolumab showed antitumor activity across AS subtypes in advanced AS patients previously treated with taxanes [96]. However, a recent trial combining the anti‐Endoglin antibody, carotuximab with pazopanib did not improve outcomes [106]. As the molecular landscape of AS is defined for each patient, more tailored therapeutic approaches for TKIs should be elucidated. As an example, in one case report, an AS patient with *KDR* and *FLT4* amplification did not respond to sorafenib, but a strong response was observed with pazopanib suggesting dual *FLT4* and *KDR* amplified AS patients may be more amenable for pazopanib treatment than other TKIs [107]. Furthermore, FLT4 IHC may aid in identifying patients with gene amplification that are amenable for targeted therapy [108]. While further studies are required these types of observations are necessary for defining the molecular features that can dictate therapeutic responses.

*PIK3CA* encodes the p110α protein, the catalytic subunit of phosphatidylinositol 3‐kinase (PI3K). PI3K‐mediated phosphorylation triggers the activation of PI3K pathway, which is crucial for fundamental cell activities [109]. The enrichment of *PIK3CA* mutations in primary breast AS was first reported by Painter et al. [26] using data from the Angiosarcoma Project and has been subsequently confirmed by other studies [3, 7, 10, 33, 110, 111]. *PIK3CA* mutations are associated with poor prognosis in patients with primary breast AS [33]. Beyond primary breast AS, *PIK3CA* is a highly mutated gene in other types of breast malignancies indicating its significant role across different types of breast cancer. Currently, there are two FDA‐approved PIK3CA inhibitors. Alpelisib is an α‐specific PI3K inhibitor that selectively inhibits p110α [112, 113, 114]. Its efficacy has been demonstrated in mouse models [114] and breast carcinoma patients [115] with *PI3KCA* mutations. Although canine *PIK3CA* mutant HSA cell lines indicate partial resistance to alpelisib [89], in the SOLAR‐1 trial, a randomized, phase 3 study, alpelisib plus fulvestrant treatment resulted in extended progression‐free survival and an improved response rate in breast cancer patients with *PIK3CA* mutation compared to placebo plus fulvestrant [115]. Copanlisib is a pan‐PI3K inhibitor that targets all four isoforms of class I PI3K and has shown effectiveness against solid tumors [116]. Evaluating these inhibitors in primary breast AS patients harboring *PIK3CA* mutations would be compelling.

*PLCG1* (phospholipase C gamma) is identified as the third most common mutated gene in primary breast AS. PLCG1 is activated by receptor tyrosine kinases and is essential for signaling cascades downstream of these receptors through mediating the production of the secondary messenger molecules diacylglycerol (DAG) and inositol 1,4,5‐trisphosphate (IP3) [117]. *PLCG1*‐*R707Q* has been reported as a hotspot mutation in AS, which causes constitutive activation of PLCG1 independently of receptor tyrosine kinases [49]. Expression of *PLCG1*‐*R707Q* mutation in endothelial cells increased activity of the c‐RAF/MEK/ ERK1/2 pathway and enhanced cell migration and invasion [49]. Interestingly *PLCG1*‐*R707Q* also arose in a sunitinib resistant hepatic AS patient sample suggesting activation of PLCG1 as a potential VEGFR TKI resistance mechanism [118]. Studies on PLCG1 targeting lipase‐independent activities indicate therapies such as SHP2 inhibitors have shown efficacy in colorectal cancer cells with high levels of PLCG1 [119]. Similar studies and approaches to test PLCG1 inhibition in AS are warranted based on its high rate of alteration in tumors. Furthermore, *PLCG1* mutational status may be useful as a biomarker for potential sensitivity or resistance to VEGFR‐based therapies.

CRK‐like (*CRKL*) is the 11th most altered gene in AS (Fig. 1) and the 3rd in HN‐AS (Fig. 2A). CRKL is an SH2/SH3 domain‐containing adaptor protein that is expressed ubiquitously and is involved in several biological processes, including cytoskeletal changes, cell proliferation, adhesion, migration, differentiation, and phagocytosis [120]. Multiple human cancers demonstrate knockdown of CRKL signaling reduces tumor growth in various cancer cell lines [121, 122, 123]. Although the role of CRKL has been studied in other cancers, its role has not been studied in AS. No CRKL inhibitors exist to date, however, their development may be of particular interest, especially in HN‐AS where it is one of the most frequently altered genes. Furthermore, since HN‐AS patients may be more amenable for immunotherapies, CRKL expression is predictive of anti‐PD1 responses in melanoma and could be a similar biomarker in AS [124]. CRKL studies in AS may be warranted in order to determine if the reported amplification is oncogenic and represents a dependency that can be therapeutically targeted.

MiRNAs are short, non‐coding RNAs that regulate gene expression by binding to the 3′UTR of transcripts, mediating degradation or translational repression [125]. Because miRNAs are essential for many biological processes, dysregulation can contribute to a myriad of human diseases, including cancer. The *Dicer1* mouse model indicates that endothelial miRNA loss drives AS development [66, 126]. In addition, many miRNAs, such as miR‐497 [61, 65], have been reported to be tumor suppressive in AS, and some studies have identified *Dicer1* mutations in patient tumors [6, 61, 62, 63, 64]. Taken together, this suggests that pharmacologic enhancement of miRNA biogenesis may be a viable therapeutic strategy to treat AS. Enoxacin is a fluoroquinolone antibiotic that can enhance pre‐miRNA to mature miRNA processing in mammalian cells, seemingly through increasing the affinity of pre‐miRNAs to TAR RNA binding protein (TRBP) [127]. Enoxacin has shown preclinical efficacy in a variety of cancers, including Ewing sarcoma [128], colorectal cancer [127], and prostate cancer [129], and thus may be a promising approach for AS. Furthermore, therapeutic delivery of miRNA mimics or anti‐miRNAs may be an additional therapeutic opportunity and have shown some promise in a preclinical setting [130, 131, 132].

Over the past few decades, immunotherapy has emerged as a promising avenue in cancer treatment. This approach includes various strategies, including immune checkpoint inhibitors, CAR‐T cell therapy, and cancer vaccines [133]. Recent advancements have demonstrated the remarkable potential of immunotherapies across a spectrum of cancer types, including soft tissue sarcomas [134], and have been used both as a monotherapy, or in combination with other therapies to boost efficacy [135]. Notably, in the context of HN‐AS, some reports have revealed the exciting potential to leverage immunotherapy, particularly in HN‐AS tumors with high TMB and UV‐damage signature [3, 9]. High TMB has been reported to be a biomarker for immune checkpoint therapies [9]. Indeed, anti‐PD1 therapies resulted in a remarkably durable response for HN‐AS patients with these signatures [26, 136]. Furthermore, ipilimumab and nivolumab combination demonstrated responses in three out of five scalp AS patients in the Dual anti‐CTLA‐4 and Anti‐PD‐1 in Rare Tumors (DARTS1609) trial [137]. In addition, a recent trial combining the anti‐PD1 antibody, nivolumab with paclitaxel showed potential benefit in some HN‐AS patients [96]. This demonstrates the exciting opportunity to leverage immunotherapy in this AS subtype.

For patients with co‐occurring mutations, the combination of targeted medicine is likely to have better efficacy than a single monotherapy. Additionally, some AS patients develop resistance to traditional VEGF pathway inhibitors with *FLT4* and *PLCG1* alterations reported to contribute to this resistance [118, 138]. For those patients, subsequent targeted therapy that specifically inhibits the gene mainly responsible for treatment resistance might be beneficial for re‐sensitizing tumors to treatment. Alternatively, targeting multiple pathways concurrently could be efficacious. Indeed, preclinical studies with combined MEK and VEGFR inhibition showed promise *in vivo* [139]. Other studies in mouse models suggest the combination of mTOR and MEK inhibition is promising [60]. Finally, a recent trial combining a multi‐TKI with immunotherapy exhibited activity across subtypes in advanced AS tumors [96]. Thus, as targeted‐personalized therapies make their way into the clinic the genomic landscape should direct the optimized treatment approaches that enhances outcomes for patients.

## 6 Conclusions and future perspectives

Angiosarcoma is a devastating disease with a low survival rate. There have been several independent studies that have reported genomic alterations in patient AS. However, because AS is rare and highly heterogeneous, the most highly altered genes can differ between cohorts. By compiling and summarizing findings from these studies, this review provides a comprehensive view of the predominant genetic alterations in AS overall, and how these alterations differ based on etiologic and anatomic subtypes, including head and neck, breast, secondary, and cardiac AS. Furthermore, in this work, we summarize the available preclinical models for AS and what is on the horizon for novel therapeutics.

*TP53* and *POT1* remain the most mutated in HN‐AS, with *CRKL* being the third most altered gene. In primary breast AS *KDR*, *PIK3CA*, and *PLCG1* are the most frequently mutated genes. Although *KDR* and *PIK3CA* alterations are observed in overall AS patients, their incidence is nearly four and five times greater in primary breast AS, respectively. Finally, frequent *POT1* and *KDR* mutations have been identified in cardiac AS. This illustrates the challenge in making progress in AS, the disease is already rare, and within this rare disease, the subtypes tend to be quite different, with likely variable responses to therapies, making it difficult to find targeted treatments that work broadly.

Having a good understanding of genetic drivers of AS allows for the development of disease models. Many animal models of AS utilize commonly mutated genes, such as *TP53*, *POT1*, and *RAS*. However, there are many common genomic alterations that are not represented in animal models. For example, animal models that utilize *PIK3CA*, *CRKL*, *PLCG1* do not yet exist. Furthermore, models driven by alterations that tend to be specific to certain AS subtypes may allow for models that recapitulate each subtype and allow researchers to better interrogate the underlying biology. This progress is imperative for improving survival outcomes for this rare, devastating disease.

## Conflict of interest

The authors declare no conflict of interest.

## Author contributions

JAH conceived the work. JAH, AB, and BL were responsible for supervision. JAH, AB, BL, and LEG reviewed, analyzed, and interpreted the data, drafted and edited the manuscript, and approved the final manuscript.
